# Chloroplast C-to-U editing, regulated by a PPR protein BoYgl-2, is important for chlorophyll biosynthesis in cabbage

**DOI:** 10.1093/hr/uhae006

**Published:** 2024-01-10

**Authors:** Bin Zhang, Yuankang Wu, Shoufan Li, Wenjing Ren, Limei Yang, Mu Zhuang, Honghao Lv, Yong Wang, Jialei Ji, Xilin Hou, Yangyong Zhang

**Affiliations:** State Key Laboratory of Crop Genetics and Germplasm Enhancement, College of Horticulture, Nanjing Agricultural University, Nanjing 210095, China; State Key Laboratory of Vegetable Biobreeding, Institute of Vegetables and Flowers, Chinese Academy of Agricultural Sciences, Beijing 100081, China; State Key Laboratory of Crop Genetics and Germplasm Enhancement, College of Horticulture, Nanjing Agricultural University, Nanjing 210095, China; State Key Laboratory of Vegetable Biobreeding, Institute of Vegetables and Flowers, Chinese Academy of Agricultural Sciences, Beijing 100081, China; State Key Laboratory of Vegetable Biobreeding, Institute of Vegetables and Flowers, Chinese Academy of Agricultural Sciences, Beijing 100081, China; State Key Laboratory of Crop Genetics and Germplasm Enhancement, College of Horticulture, Nanjing Agricultural University, Nanjing 210095, China; State Key Laboratory of Vegetable Biobreeding, Institute of Vegetables and Flowers, Chinese Academy of Agricultural Sciences, Beijing 100081, China; State Key Laboratory of Vegetable Biobreeding, Institute of Vegetables and Flowers, Chinese Academy of Agricultural Sciences, Beijing 100081, China; State Key Laboratory of Vegetable Biobreeding, Institute of Vegetables and Flowers, Chinese Academy of Agricultural Sciences, Beijing 100081, China; State Key Laboratory of Vegetable Biobreeding, Institute of Vegetables and Flowers, Chinese Academy of Agricultural Sciences, Beijing 100081, China; State Key Laboratory of Vegetable Biobreeding, Institute of Vegetables and Flowers, Chinese Academy of Agricultural Sciences, Beijing 100081, China; State Key Laboratory of Vegetable Biobreeding, Institute of Vegetables and Flowers, Chinese Academy of Agricultural Sciences, Beijing 100081, China; State Key Laboratory of Crop Genetics and Germplasm Enhancement, College of Horticulture, Nanjing Agricultural University, Nanjing 210095, China; State Key Laboratory of Vegetable Biobreeding, Institute of Vegetables and Flowers, Chinese Academy of Agricultural Sciences, Beijing 100081, China

## Abstract

Leaf color is an important agronomic trait in cabbage (*Brassica oleracea* L. var. *capitata*), but the detailed mechanism underlying leaf color formation remains unclear. In this study, we characterized a *Brassica oleracea yellow-green leaf 2* (*BoYgl-2*) mutant 4036Y, which has significantly reduced chlorophyll content and abnormal chloroplasts during early leaf development. Genetic analysis revealed that the yellow-green leaf trait is controlled by a single recessive gene. Map-based cloning revealed that *BoYgl-2* encodes a novel nuclear-targeted P-type PPR protein, which is absent in the 4036Y mutant. Functional complementation showed that *BoYgl-2* from the normal-green leaf 4036G can rescue the yellow-green leaf phenotype of 4036Y. The C-to-U editing efficiency and expression levels of *atpF*, *rps14*, *petL* and *ndhD* were significantly reduced in 4036Y than that in 4036G, and significantly increased in *BoYgl-2* overexpression lines than that in 4036Y. The expression levels of many plastid- and nuclear-encoded genes associated with chloroplast development in *BoYgl-2* mutant were also significantly altered. These results suggest that *BoYgl-2* participates in chloroplast C-to-U editing and development, which provides rare insight into the molecular mechanism underlying leaf color formation in cabbage.

## Introduction

The chloroplast is a crucial organelle for photosynthesis and provides necessary energy for plant growth and development. Chlorophyll is the critical pigment responsible for absorbing and transferring light energy to the photosynthetic system. Leaf color mutants are usually caused by impaired chloroplasts and blocked chlorophyll synthesis. Leaf color variations affect the photosynthetic rate, resulting in stunted plant growth and reduced yields. Leaf color mutants can be used to rapidly identify variety purity in crop hybrid breeding and understand plant photosynthetic mechanisms, chlorophyll anabolic pathways, and gene regulatory networks [1–5].

Pentatricopeptide repeat (PPR) is a large protein family that participates in post-transcriptional RNA modification, such as RNA editing, RNA processing, RNA translation, RNA stability and RNA splicing [6–10]. PPR proteins can be classified into P and PLS subfamilies and play essential roles in chloroplast development [11]. In *Arabidopsis*, Wang et al. identified the P-type PPR protein ECD2; *ECD2* RNAi lines had an albino cotyledon phenotype. Further analysis revealed that *ECD2* is related to chloroplast gene expression and group II intron splicing [12]. In soybean, Feng et al. identified a PLS-type PPR protein GmPGL2, and plants with mutant *GmPGL2* exhibited pale-green leaves. *GmPGL2* is essential for C-to-U editing of *ndhB*, *ndhD*, *rps16*, *ndhF*, and *ndhE*, *rps18* genes in chloroplasts [13]. In rice, Lan et al. identified a young leaf white stripe (*ylws*) mutant. *YLWS* encodes a P-type PPR protein, which mutation causes defects in chloroplast RNA editing of *ndhA*, *rps14* and *ndhB*, and RNA splicing of *ndhA*, *rps12*, *atpF* and *rpl2* genes [14]. Huang et al. identified a PLS-DYW subfamily PPR protein OsPPR16, which is required for RNA editing of *rpoB*-545 in rice plastids [15]. In maize, a *qKW9* mutant with smaller ears and fewer kernels was identified. *qKW9* encodes a PLS-type PPR protein that affects chloroplast C-to-U editing of *ndhB* and photosynthesis [16].

Chromosomal deletion is a classic type of structural variation, and several chromosome fragment deletions are associated with crop-specific phenotypes. Li et al. identified a 13.96-kb chromosomal fragment deletion in watermelon. Through expression and co-segregation marker analysis, two genes *Cla97C02G045390* and *Cla97C02G045400* in the chromosomal deletion region were found to be responsible for the seed size in watermelon [17]. In *Brassica napus*, Zhang et al. localized the delay-green leaf gene *BnaA02.YTG1* to a 9.9-kb region and found that the fragment was deleted in the *ytg* mutant by sequence analysis. By functional analysis, the *BnaA02g10480D* gene in the chromosomal deletion region was found to control the delay-green leaf phenotype and participate in the chloroplast RNA editing in rapeseed [18]. In *Cucurbita pepo*, Zhu et al. mapped the dark-green stem color gene *CpDsc-1* to a 65.2-kb interval, and a 14-kb chromosomal fragment deletion between *Cp4.1LG15g03360* and *Cp4.1LG15g03420* genes was identified in the candidate region. Expression and co-dominant marker analysis indicated that *Cp4.1LG15g03420* may be the main cause of the dark-green stem phenotype in zucchini [19]. In *Arabidopsis*, a 14-kb deletion on chromosome 3 was identified in the T-DNA mutant SALK_008491, and the loss of both *NHD1* and *PGDH3* causes SALK_008491 highly sensitive to dynamic light stress [20].

In this study, we identified a new spontaneous yellow-green leaf mutant *BoYgl-2* in cabbage. Using BC_1_ and F_2_ populations, the *BoYgl-2* gene was fine mapped to a tiny region by BSA-seq and linkage analysis, and a large chromosomal deletion was detected in the *BoYgl-2* locus. *BoYgl-2* encodes a nuclear-targeted P-type PPR protein that affects chloroplast RNA editing. This finding lays a foundation for revealing the molecular mechanism underlying leaf color formation in cabbage.

## Results

### Characterization of the *BoYgl-2* mutant

**Figure 1:**
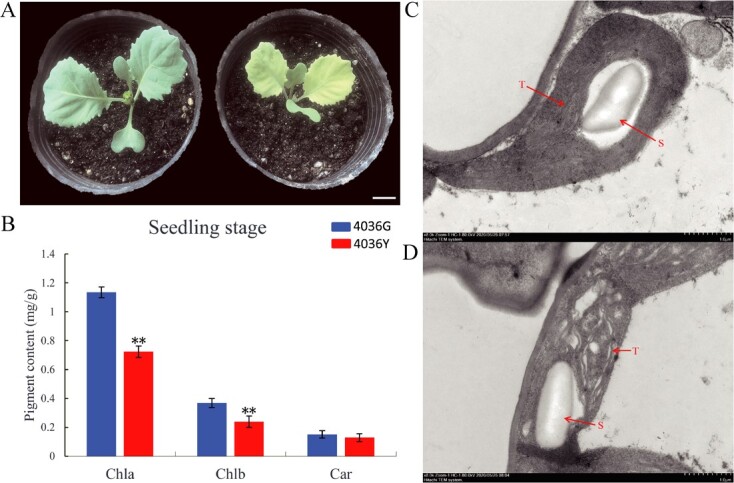
Phenotypic characterization of the *BoYgl-2* mutant. **(A)** 4036G with normal-green leaves (left) and 4036Y with yellow-green leaves (right). Bar = 2.5 cm. **(B)** Pigment contents in the leaves of 4036G and 4036Y at the seedling stage. Error bars represent the standard errors of three biological replicates (Student’s *t*-test: **P < 0.01). **(C)** Chloroplast ultrastructure in the seedling leaves of 4036G. **(D)** Chloroplast ultrastructure in the seedling leaves of 4036Y. T, thylakoid membrane; S, starch grain.

Compared with 4036G (normal-green leaf), the 4036Y mutant exhibited a yellow-green leaf phenotype at seedling stage and returned to normal-green leaf at mature stage (Fig. 1A). The Chl a and Chl b contents were significantly reduced in 4036Y than that of 4036G at the seedling stage, but had no significant difference at the mature stage (Fig. 1B, Supplemental Fig. S1). To investigate the effect of the *BoYgl-2* mutation on chloroplast biogenesis, the chloroplast ultrastructures of the leaves of 4036G and 4036Y seedlings were observed by transmission electron microscopy (TEM). The chloroplasts in 4036G leaves exhibited well-structured and well-organized thylakoid membranes (Fig. 1C). However, in the 4036Y mutant, the chloroplasts lacked organized thylakoid membranes (Fig. 1D). These results suggest that *BoYgl-2* is involved in Chl biosynthesis and chloroplast development.

### Fine mapping of the *BoYgl-2* gene

To identify the *BoYgl-2* gene, the 4036Y mutant was crossed with 4036G. All the leaves of the F_1_ plants were normal green. The F_2_ population comprised 2508 individuals, with 1885 normal-green leaf and 623 yellow-green leaf individuals, the segregation ratio is 3:1. All BC_1_P_1_ individuals showed normal-green leaf. The BC_1_P_2_ population contained 653 yellow-green leaf and 659 normal-green leaf individuals, with a segregation ratio of 1:1 (Table 1). These results indicate that the yellow-green leaf phenotype is controlled by a single recessive nuclear gene.

**Table 1:** Genetic analysis of the yellow-green leaf trait in BC_1_ and F_2_ populations

Populations	Total plant number	Number of normal–green leaf plants*	Number of yellow–green leaf plants*	Expected ratio	χ^2a^
F_1_	16	16	0	-	-
F_2_	2508	1885	623	3:1	0.03
BC_1_P_1_	120	120	0	-	-
BC_1_P_2_	1312	659	653	1:1	0.03

^*^Normal-green and yellow-green leaf plants were identified at the seedling stage by visual inspection

^a^χ^2^ >χ^2^_0.05_ = 3.84 was considered significant

**Figure 2:**
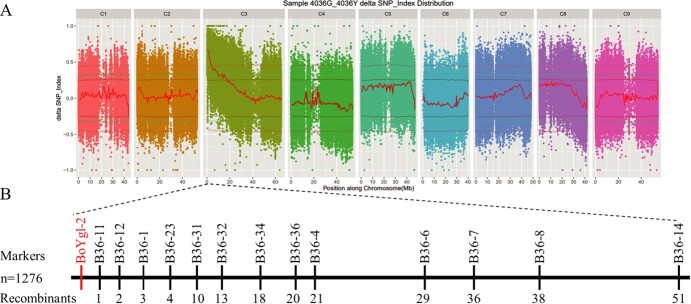
Fine mapping of the *BoYgl-2* gene. **(A)** Plot of the Δ(SNP-index) value obtained from the two bulks. The top red line indicates the threshold line (99% confidence level). The x-axis is the position of nine chromosomes and the y-axis is the Δ(SNP-index) value. **(B)** Linkage map of the *BoYgl-2.* InDel marker B36-11 was closely linked to the *BoYgl-2* gene.

BSA-seq analysis was performed to preliminarily map the *BoYgl-2* gene. The highest peak region (*P*<0.01), which contains 5.36 Mb (0–5.36 Mb) on chromosome 3 according to the cabbage reference genome (TO1000), was taken as the candidate interval associated with *BoYgl-2* (Fig. 2A). To fine map the *BoYgl-2*, 13 InDel markers with polymorphism between the parents were developed within the 5.36-Mb candidate region and then used to analyze a total of 1276 recessive individuals (yellow-green leaf) from the BC_1_P_2_ and F_2_ populations. A linkage map consisting of 13 InDel markers (Supplementary Table S1) was constructed. The InDel marker B36-11 was found to be closely linked to *BoYgl-2*, with a genetic distance of 0.1 cM. Based on Based on the location of marker in the reference genome, *BoYgl-2* was ultimately mapped to a 232-kb region (C03: 0 bp-232,068 bp) (Fig. 2B).

### A 162-kb chromosomal deletion was identified at the *BoYgl-2* locus

**Figure 3:**
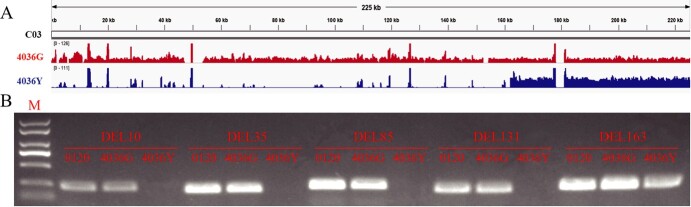
Identification of the chromosomal deletion at the *BoYgl-2* locus. **(A)** Visual analysis of the sequence reads of 4036G and 4036Y at the *BoYgl-2* locus by IGV. **(B)** Amplicons of the markers DEL10, DEL35, DEL85, DEL131 and DEL163 in 0120, 4036G and 4036Y. M represents the DNA ladder.

To identify the candidate gene for *BoYgl-2*, visual analysis of reads in the candidate interval was performed by IGV (Integrative Genomics Viewer) using the resequencing data of 4036G and 4036Y. A 162-kb chromosome deletion (0 bp-161,975 bp) was detected at the *BoYgl-2* locus in 4036Y mutant, reads with high sequencing depth in this region were genome highly repetitive sequences (Fig. 3A). Subsequently, five markers, DEL10, DEL35, DEL85, DEL131, and DEL163 (at positions 10 kb, 35 kb, 85 kb, 131 kb, and 163 kb, respectively), were developed based on the TO1000 genome. These markers were then used to verify the parents and another cabbage normal-green leaf inbred line, 0120. The results showed that the target bands of all the markers could be amplified in 0120 and 4036G, but only the target band of DEL163 (outside the chromosomal deletion fragment) could be amplified in 4036Y (Fig. 3B), verifying the large chromosomal terminal deletion at the *BoYgl-2* locus in the 4036Y mutant.

### Functional verification of *BoYgl-2*

Based on the cabbage reference genome (TO1000), 53 genes were identified within the 232-kb candidate interval (Supplementary Table S2). According to the comparative genomic annotation in *A. thaliana* (TAIR), only one gene, *Bo3g001140*, was strongly related to the formation of leaf color. *Bo3g001140* is a homolog of the *AT1G02420* gene in *Arabidopsis*, which encodes a pentatricopeptide repeat (PPR) protein involved in chloroplast RNA processing and is located in the chromosomal deletion region. Many PPR mutants exhibit a yellow-green leaf or albino phenotype [12–14, 21]. Thus, we designated *Bo3g001140* as the candidate gene for *BoYgl-2*.

**Figure 4:**
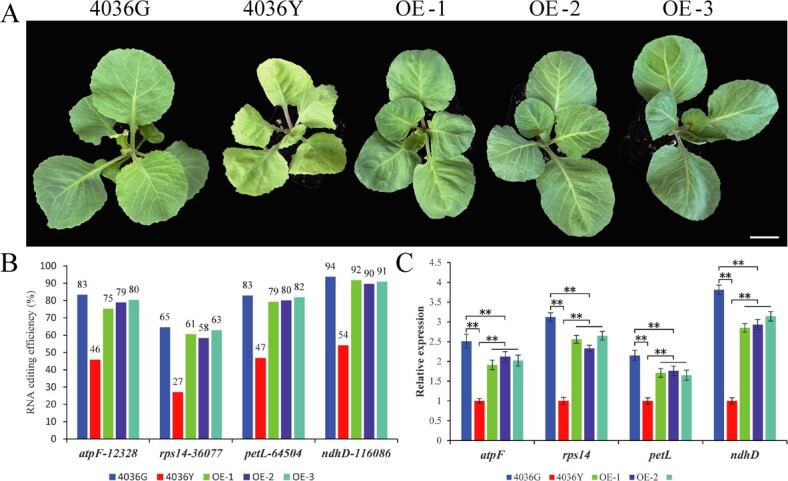
Functional analysis of the *BoYgl-2* gene, RNA editing and expression analysis of *atpF*, *rps14*, *petL* and *ndhD*. **(A)** Phenotypes of *BoYgl-2* overexpression lines at the seedling stage. Bar = 3 cm. **(B)** C-to-U editing efficiency of *atpF*, *rps14*, *petL* and *ndhD* in 4036G, 4036Y and *BoYgl-2* overexpression lines at the seedling stage. **(C)** Expression levels of *atpF*, *rps14*, *petL* and *ndhD* in 4036G, 4036Y and *BoYgl-2* overexpression lines at the seedling stage. Error bars represent the standard errors of three biological replicates (Student’s *t*-test: **P < 0.01).

To test whether *Bo3g001140* is responsible for the *BoYgl-2* phenotype, we transformed the full-length CDS of *Bo3g001140*, driven by the CaMV 35S promoter, into 4036Y mutants, and obtained three independent overexpressing transgenic T_1_ lines. All the overexpression lines showed a normal-green leaf phenotype similar to 4036G (Fig. 4A), and qRT-PCR analysis showed that the expression level of *Bo3g001140* was significantly higher in overexpression lines than that in 4036Y mutant (Supplemental Fig. S2). These results indicate that *Bo3g001140* is the *BoYgl-2* gene controlling leaf color formation in cabbage.

### *BoYgl-2* encodes a nuclear-localized P-type PPR protein

**Figure 5:**
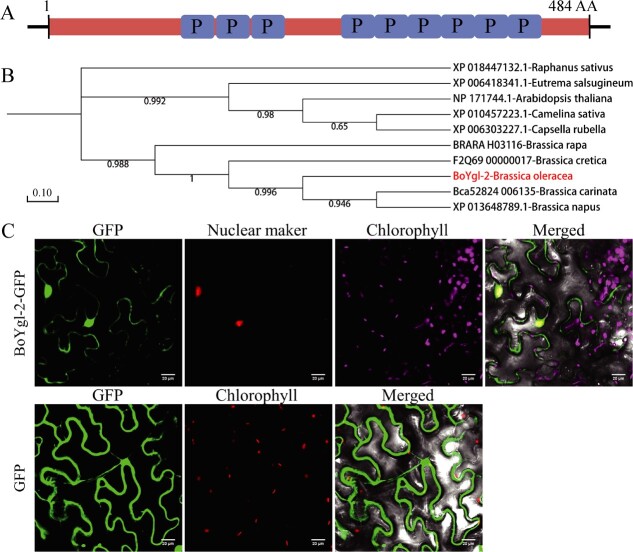
Phylogenetic analysis and subcellular localization of BoYgl-2. **(A)** Schematic diagram of the BoYgl-2 protein with nine PPR motifs. Blue boxes represent P-type repeats. **(B)** Phylogenetic tree of the BoYgl-2 protein and its nine homologues from other cruciferous species. Numbers represent bootstrap values. **(C)** Subcellular localization of the BoYgl-2:GFP fusion protein. Bar = 20 μm.

Sequence analysis revealed that *BoYgl-2*, which encodes a putative protein of 484 amino acids, contained nine PPR motifs and belonged to the P-type subfamily according to the TPRpred database (https://toolkit.tuebingen.mpg.de/tools/tprpred) (Fig. 5A). Next, a phylogenetic tree of the BoYgl-2 protein and its nine homologues from other cruciferous species was constructed to analyze their evolutionary relationship. The results showed that BoYgl-2 was conserved in these homologous and shared a closer relationship with *Brassica cretica*-F2Q69_00000017 (98.55%) and *Brassica carinata*-Bca52824_006135 (97.73%), indicating that they may have originated from the same ancestor gene (Fig. 5B). To determine the subcellular localization of BoYgl-2, we performed a transient expression assay in tobacco leaves. The GFP fluorescence of BoYgl-2:GFP fusion protein was colocalized with the nuclear marker fluorescence (Fig. 5C). The results demonstrated that BoYgl-2 is a nuclear-localized PPR protein.

### Chloroplast RNA editing is impaired in the *BoYgl-2* mutant

Many PPR proteins participate in chloroplast RNA editing [13, 15, 22, 23]. We determined whether this was the case for BoYgl-2. By RNA-seq analysis, 29 C-to-U editing sites were identified in the chloroplast of the parental lines. Editing-specific markers were then used to examine the C-to-U editing efficiency of the 29 editing sites in 4036G and 4036Y at the seedling stage. Five editing sites (*rpoC1*-21382, *accD*-57118, *psbF*-62793, *rps12*-*clpP*-68694, and *ndhD*-115201) showed a less than 10% increase, and five editing sites (*rpoB*-23461, *rpoB*-25327, *lhbA*-34880, *rpoA*-77634, and *rpl2*-exon2-152763) exhibited no difference in C-to-U editing efficiency in 4036Y. The C-to-U editing efficiency of the remaining 19 editing sites was reduced to varying degrees in 4036Y, among which the *atpF*-12328, *rps14*-36077, *petL*-64504, and *ndhD*-116086 were reduced by more than 36% (Table 2). In *BoYgl-2* overexpression lines, the C-to-U editing efficiency of *atpF*-12328, *rps14*-36077, *petL*-64504 and *ndhD*-116086 was significantly increased and recovered to be similar to 4036G (Fig. 4B). In addition, qRT-PCR analysis showed that the expression levels of *atpF*, *rps14*, *petL* and *ndhD* were significantly decreased in 4036Y than that in 4036G, and significantly increased in *BoYgl-2* overexpression lines than that in 4036Y mutant (Fig. 4C). These results indicate that *BoYgl-2* is important for improving the C-to-U editing efficiency of *atpF*, *rps14*, *petL* and *ndhD* in cabbage chloroplast.

**Table 2:** Chloroplast RNA editing efficiency analysis in 4036G and 4036Y.

Gene	Region	Position	4036G	4036Y	Difference	Nucleotide change	Amino acid change
*rps16*	intronic	5515	62.50%	58.33%	-4.17%	/	/
*atpF*	exonic	12328	83.33%	45.83%	-37.50%	C->T	Pro-Leu
*rpoC1*	exonic	21382	68.75%	78.72%	9.97%	C->T	Ser-Leu
*rpoB*	exonic	23461	100.00%	100.00%	0.00%	C->T	Ser-Leu
*rpoB*	exonic	25327	89.58%	89.58%	0.00%	C->T	Ser-Leu
*rpoB*	exonic	25342	89.58%	83.33%	-6.25%	C->T	Ser-Leu
*lhbA*	exonic	34880	100.00%	100.00%	0.00%	C->T	Ser-Leu
*rps14*	exonic	36077	64.58%	27.08%	-37.50%	C->T	Pro-Leu
*accD*	exonic	56509	97.92%	93.75%	-4.17%	C->T	Ser-Leu
*accD*	exonic	57118	91.67%	95.83%	4.17%	C->T	Pro-Leu
*psbF*	exonic	62793	89.58%	91.67%	2.08%	C->T	Ser-Phe
*petL*	exonic	64504	82.98%	46.81%	-36.17%	C->T	Pro-Leu
*rps12*-*clpP*	intergenic	68694	43.75%	62.50%	18.75%	/	/
*clpP*	exonic	68755	60.42%	45.83%	-14.58%	C->T	His-Tyr
*rpoA*	exonic	77634	100.00%	100.00%	0.00%	C->T	Ser-Phe
*ndhB*	exonic	94173	81.25%	52.08%	-29.17%	C->T	His-Tyr
*ndhB*	exonic	94556	68.75%	54.17%	-14.58%	C->T	Ser-Leu
*ndhB*	exonic	94598	62.50%	41.67%	-20.83%	C->T	Ser-Leu
*ndhB*	exonic	95361	77.08%	70.83%	-6.25%	C->T	Ser-Phe
*ndhB*	exonic	95958	91.67%	89.58%	-2.08%	C->T	Ser-Leu
*ndhF*	exonic	111249	89.58%	79.17%	-10.42%	C->T	Ser-Leu
*ndhD*	exonic	114778	93.75%	87.50%	-6.25%	C->T	Ser-Leu
*ndhD*	exonic	115201	77.08%	79.17%	2.08%	C->T	Pro-Leu
*ndhD*	exonic	115210	95.83%	93.75%	-2.08%	C->T	Ser-Leu
*ndhD*	exonic	115414	95.83%	93.75%	-2.08%	C->T	Ser-Leu
*ndhD*	exonic	115705	100.00%	95.83%	-4.17%	C->T	Ser-Leu
*ndhD*	exonic	116086	93.75%	54.17%	-39.58%	C->T	Thr-Met
*ndhG*	exonic	117780	97.92%	87.50%	-10.42%	C->T	Ser-Phe
*rpl2*-exon2	splicing	152763	100.00%	100.00%	0.00%	/	/

### Expression analysis of chloroplast-related genes

**Figure 6:**
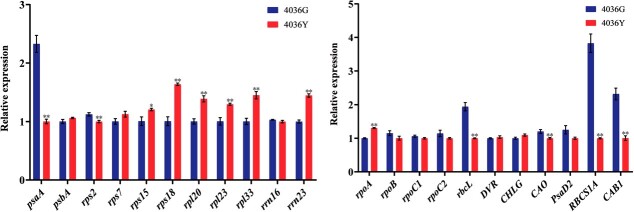
Expression patterns of genes associated with chloroplast development in 4036G and 4036Y. *BoActin* served as the equal loading control. Error bars represent the standard errors of three biological replicates (Student’s *t*-test: **P < 0.01).

Many studies have shown that PPR proteins are involved in chloroplast development [4, 13, 21, 23]. We analyzed the expression patterns of chloroplast-related genes in 4036G and 4036Y. The expression levels of the PEP-dependent genes *psaA* and *rbcL* and ribosome gene *rps2* were significantly reduced, and the expression levels of the NEP-dependent gene *rpoA* and ribosome genes *rps15*, *rps18*, *rpl20*, *rpl23*, *rpl33*, and *23S rRNA* were significantly increased. However, the expression levels of other chloroplast-encoded genes *psbA* (PEP-dependent), *rpoB*, *rpoC1*, *rpoC2* (NEP-dependent), *rps7*, and *16S rRNA* (ribosomal) did not differ significantly between 4036Y and 4036G. The expression levels of the photosynthesis-associated genes, *RBCS1A* and *CAB1*, and the Chl biosynthesis gene, *CAO*, were significantly down-regulated, while the expression levels of other nuclear-encoded genes *PsaD2* (photosynthetic), *DVR*, and *CHLG* (Chl biosynthetic) were not significantly altered in 4036Y compared with 4036G (Fig. 6). These results suggest that the expression patterns of genes related to chloroplast development, Chl biosynthesis and photosynthesis were significantly affected in the *BoYgl-2* mutant.

## Discussion

Most PPR proteins are localized and function in chloroplasts or mitochondria. In *Arabidopsis*, rice and maize, some chloroplast-targeted PPR proteins have been identified, which are related to pale-green, albino, pale-yellow, and white-striped leaf formation [4, 14, 15, 21, 22, 24]. And several mitochondrion-localized PPR proteins have been identified to be associated with leaf albino, pale-green and dark curling and seed development [25–28]. In addition, very few PPR proteins have been reported to be localized in the nucleus. Hao et al. identified a rice nuclear-targeted PPR protein OsNPPR1 that is essential for mitochondrial function and endosperm development [29]. In *Arabidopsis*, a nuclear-localized PPR protein GRP23, and a mitochondrion and nuclear dual-localized PPR protein were identified, which are essential for early embryo development [30, 31]. In this study, we identified the first nuclear-targeted P-type PPR protein BoYgl-2 in cabbage, which is important for chloroplast development and chlorophyll biosynthesis, providing novel insights into the molecular mechanism underlying leaf color formation in cabbage.

PPR proteins are crucial for the normal activities of chloroplasts and mitochondria. Many *PPR* gene mutations result in variable leaf color phenotypes. In rice, Chen et al. identified a PLS-type PPR protein, PGL12. A single-base mutation (C to T) in the *pgl12* gene generates a premature stop codon, which severely affects the 16S rRNA processing and plastid *ndhA* RNA splicing, resulting in the pale-green leaf phenotype of *pgl12* mutant [21]. Wang et al. identified a P-type PPR protein WSL4. A 2-bp deletion in the *WSL4* gene, generating a premature stop codon, causes defects in chloroplast RNA editing of *rpoB*, and RNA splicing of *atpF*, *ndhA*, *rpl2*, and *rps12* genes, which results in the white-stripe leaf phenotype of *WSL4* mutant [23]. In soybean, Feng et al. identified a pale-green leaf mutant *Gmpgl2*. A single-base deletion in the *Gmpgl2* gene produces a truncated protein that lacks portion of the E2 and E+ motifs, which affects the C-to-U editing of NDH complex subunits and ribosome genes [13]. In maize, a P-type PPR protein PPR8522 was identified, and a 3.3-kb *MuDR* insertion in *PPR8522* is responsible for the *emb8522* mutant seedling albino phenotype [24]. In the present study, a nuclear-localized P-type PPR protein, BoYgl-2, was identified, which is completely absent in the 4036Y mutant. The C-to-U editing efficiency and expression levels of *atpF*, *rps14*, *petL* and *ndhD* were significantly decreased in 4036Y than that in 4036G, and significantly increased in *BoYgl-2* overexpression lines than that in 4036Y, indicating that the deletion of *BoYgl-2* impaired the C-to-U editing of *atpF*, *rps14*, *petL*, and *ndhD* and thus affected the chlorophyll biosynthesis in *BoYgl-2* mutant. BoYgl-2 is localized in the nucleus, but how it participates in C-to-U editing of chloroplast genes and affects chloroplast function in cabbage remains unclear. We speculated that BoYgl-2 may indirectly regulate chloroplast RNA editing and chlorophyll biosynthesis through a new pathway, which requires further experimental verification in the future.

Marker-assisted selection is an effective method for genetic breeding in many *B. oleracea* crops. Zhang et al. identified a lobed leaf gene *BoLMI1a* in ornamental kale. dCAPS marker DMLMI1 and co-dominant marker CMLMI1 were developed based on the *BoLMI1a* promoter variations, which can be used for marker-assisted selection of leaf shape in ornamental kale breeding [32]. Zhang et al. identified a petal color gene *BoCCD4.2* in Chinese kale. Based on the CACTA-like transposon insertion in *BoCCD4.2*, a co-dominant marker TE3 was developed that can be used for marker-assisted selection of white/yellow petal color in Chinese kale [33]. In cabbage, Han et al. identified a male-sterile gene *BoTPD1*, a *BoTPD1*-specifc marker based on the 182-bp InDel co-segregated with male sterility and can be used for marker-assisted selection [34]. Ji et al. identified a wax-deficient gene *BoCER2*. According to a G-to-A substitution in the *BoCER2* coding region, a *BoCER2*-specifc KASP marker was designed, which can be used for marker-assisted selection for glossiness [35]. In this study, we identified a yellow-green leaf gene *BoYgl-2*, and a 162-kb chromosomal deletion was identified at the *BoYgl-2* locus. Four markers, DEL10, DEL35, DEL85, and DEL131 were developed in the chromosomal deletion region that can only be amplified in normal-green leaf materials. These markers can be used for marker-assisted selection of leaf color in cabbage breeding.

## Materials and methods

### Plant materials

The 4036G (P_1_) is a cabbage inbred line with normal-green leaves; the 4036Y (P_2_) is a yellow-green leaf mutant isolated from 4036G. 4036G was crossed with 4036Y to produce F_1_ lines. F_1_ lines were self-pollinated to generate F_2_ population; the BC_1_P_1_ and BC_1_P_2_ populations were generated by backcrosses of F_1_ × 4036G and F_1_ × 4036Y, respectively. All plant materials used in this study were grown in a greenhouse (16 h light/8 h dark photoperiod; 25 °C ± 3 °C) at the Institute of Vegetables and Flowers, Chinese Academy of Agriculture Sciences (IVFCAAS, Beijing, China).

### Pigment content and transmission electron microscopy analysis

Chlorophyll and carotenoid contents were determined as previously described [18]. Fresh leaves (~0.2 g) of 4036G and 4036Y were collected at the seedling stage, and then placed in 5 ml of 80% acetone for 24 h in the dark. Chl a, Chl b and carotenoid contents were measured using a DU800 spectrophotometer (Beckman Coulter, USA) at wavelengths of 663, 645 and 470 nm, respectively. Three biological replicates were performed per sample.

Transmission electron microscopy (TEM) was performed on 4-week-old leaves of 4036G and 4036Y as previously described [5].

### BSA-seq analysis and fine mapping of the *BoYgl-2* gene

Young leaves from thirty normal-green leaf and thirty yellow-green leaf BC_1_ individuals (4-week-old) were collected to construct two bulks. High-quality genomic DNAs from the two bulks and two parental lines were extracted using the FastPure Plant DNA Isolation Mini Kit (Vazyme, Nanjing, China) to construct Illumina libraries [32], which were subsequently sequenced on an Illumina Hi-Seq 2500 sequencer by Biomarker Technologies Co., Ltd. (Beijing, China). Δ(SNP-index) analysis was performed as previously described [36].

InDel markers were designed by comparing the resequencing data from the candidate region of the 4036G and 4036Y parents. The yellow-green leaf individuals from the BC_1_P_2_ and F_2_ populations were analyzed by the markers with polymorphism between the parents. Primer design, PCR analysis, and genetic and physical map construction were performed according to the method described before [32].

### Vector construction and cabbage transformation

For the complementation experiment, the full-length CDS of *BoYgl-2* was amplified from the 4036G and cloned into the binary expression vector pBWA(V)BS-CaMV 35S. The recombinant plasmid was then transformed into 4036Y mutant by *Agrobacterium*-mediated cabbage transformation. Positive transgenic plants were identified by PCR amplification. All primers used for vector construction and identification are listed in Supplementary Table S1.

### Phylogenetic analysis and subcellular localization

Homologues of the BoYgl-2 protein were obtained from the National Center for Biotechnology Information (NCBI) database by a BLASTP search. Phylogenetic analysis was performed as described previously [32].

The full-length CDS of *BoYgl-2* without the stop codon was cloned into the green fluorescent protein (GFP) expression vector pBWA(V)HS-35S-GFP. Far red fluorescent protein (mKate) expression vector was used as the nuclear marker. The fusion construct and the mKate construct were transformed into *Agrobacterium* GV3101 and then injected into tobacco leaves as described previously [5]. GFP fluorescence signals were detected under a confocal laser-scanning microscope (Carl Zeiss, Germany).

### RNA editing analysis

Total RNA was isolated from the young leaves of 4-week-old seedlings of 4036G and 4036Y using the FastPure Universal Plant Total RNA Isolation Kit (Vazyme, Nanjing, China). cDNA was synthesized using the HiScript III 1st Strand cDNA Synthesis Kit (Vazyme, Nanjing, China). The cDNAs were subsequently used for Illumina library construction and sequencing with an Illumina Hi-Seq 4000 sequencer by Biomarker Technologies Co., Ltd. The transcripts of chloroplast genes were identified by referencing the cabbage chloroplast genome [37]. RNA editing site analysis was performed as previously reported [13].

Specific markers for RNA editing sites were designed based on the cabbage chloroplast reference genome (Supplementary Table S1). RNA editing-specific markers were then used to amplify the cDNAs from the young leaves of 4-week-old seedlings of 4036G and 4036Y. PCR products were then cloned into a T-vector using the TA/Blunt-Zero Cloning Kit (Vazyme, Nanjing, China). Fifty positive clones were selected from each sample for sequencing and RNA editing efficiency analysis.

### Expression analysis

The expression patterns of genes related to chloroplast development were analyzed by quantitative real-time PCR (qRT–PCR). A FastPure Universal Plant Total RNA Isolation Kit (Vazyme, Nanjing, China) was used to extract total RNA from 4-week-old seedling fresh leaves of 4036G and 4036Y according to the manufacturer’s instructions. cDNA was synthesized using the TIANGEN FastKing RT Kit (Tiangen, Beijing, China). qRT–PCR was carried out using the ChamQ Universal SYBR qPCR Master Mix (Vazyme, Nanjing, China) on a CFX96 Real-Time System (Bio-Rad, USA). Relative expression levels of the genes were calculated using the 2^−ΔΔCt^ method [38]. Three biological and three technical replicates were performed for all experiments. *BoActin* was used as the internal control gene. The qRT–PCR primers are listed in Supplementary Table S1.

## Supplementary Material

Web_Material_uhae006

## Data Availability

All the data generated or analyzed in this study are included in this published article and its supplementary information files. All the sequence data of the present study have been deposited in the NCBI Sequence Read Archive (SRA) database under BioProject PRJNA1015626.
