# Supplementary material for: Chloroplast C-to-U editing, regulated by a PPR protein BoYgl-2, is important for chlorophyll biosynthesis in cabbage
Source: Hortic Res. 2024 Jan 10;11(3):uhae006. doi: 10.1093/hr/uhae006 (PMC10980974; doi:10.1093/hr/uhae006)
Supplement: Web_Material_uhae006 [file web_material_uhae006.zip › Supplementary information.pdf]

Supplementary information

Figure S1. Pigment contents in the leaves of 4036G and 4036Y at the mature stage. Error bars represent the standard errors of three biological replicates, asterisks indicate statistical significance (**P < 0.01, Student's *t*-test).

Figure S2. Expression levels of *BoYgl-2* in 4036G, 4036Y and overexpression lines at the seedling stage. Error bars represent standard errors of three biological replicates. Asterisks indicate statistical significance (**P < 0.01, Student's *t*-test).

Table S1. Primer sequences of the markers used in this study.

Table S2. The 53 predicted genes in the candidate region.
