# Supplementary figures and images for: Chloroplast C-to-U editing, regulated by a PPR protein BoYgl-2, is important for chlorophyll biosynthesis in cabbage
Source: Hortic Res. 2024 Jan 10;11(3):uhae006. doi: 10.1093/hr/uhae006 (PMC10980974; doi:10.1093/hr/uhae006)

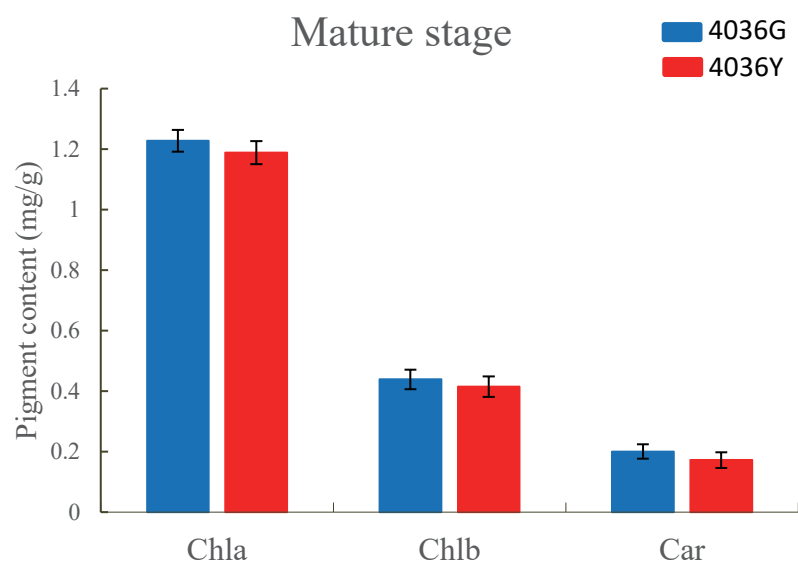

Supplement: Web_Material_uhae006 [file web_material_uhae006.zip › Figure S1.pdf]

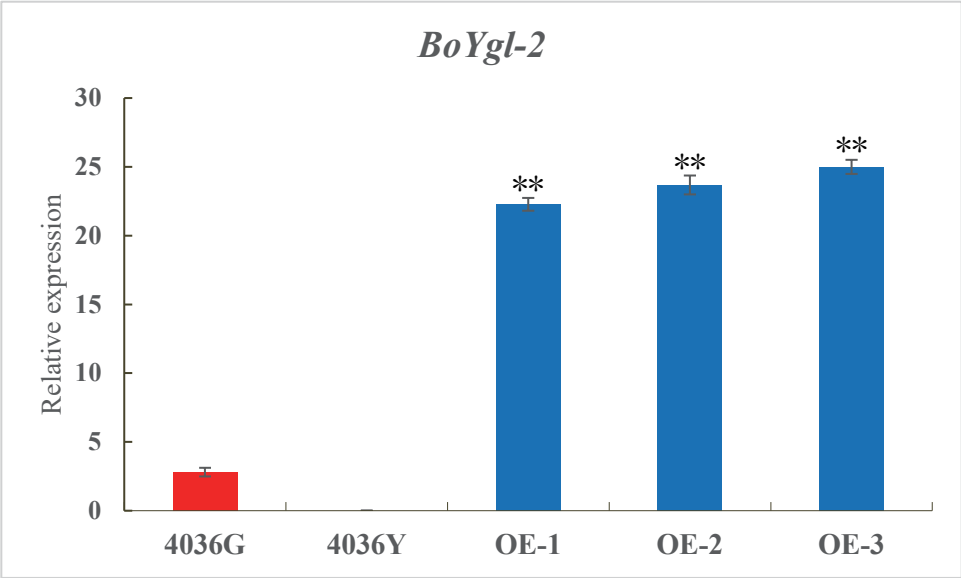

Supplement: Web_Material_uhae006 [file web_material_uhae006.zip › Figure S2.pdf]
